# Correction: Lara-Romero et al. Phylogenetic and Molecular Analysis of the Porcine Epidemic Diarrhea Virus in Mexico during the First Reported Outbreaks (2013–2017). *Viruses* 2024, *16*, 309

**DOI:** 10.3390/v18050573

**Published:** 2026-05-19

**Authors:** Rocío Lara-Romero, Rebeca Martínez-Bautista, Raúl González-Martínez, Jazmín De la Luz-Armendáriz, Irma Herrera-Camacho, Nora Rosas-Murrieta, Laura Márquez-Valdelamar, José Francisco Rivera-Benítez

**Affiliations:** 1 Posgrado en Ciencias de la Producción y de la Salud Animal, Facultad de Estudios Superiores Cuautitlán, Universidad Nacional Autónoma de México, Mexico City 04510, Mexico; lara.romero.mx@gmail.com; 2 Zoetis Swine, Mexico City 05120, Mexico; rebeca.martinez@zoetis.com; 3 Cargill, Mexico City 01210, Mexico; raul_gonzalez@cargill.com; 4 Departamento de Medicina y Zootecnia de Rumiantes, Facultad de Medicina Veterinaria y Zootecnia, Universidad Nacional Autónoma de México, Mexico City 04510, Mexico; delaluzarmendarizj@fmvz.unam.mx; 5 Laboratorio de Bioquímica y Biología Molecular, Centro de Química, Instituto de Ciencias, Benemérita Universidad Autónoma de Puebla, Puebla 72000, Mexico; irma.herrera@correo.buap.mx (I.H.-C.); nora.rosas@correo.buap.mx (N.R.-M.); 6 Laboratorio de Secuenciación Genómica de la Biodiversidad y de la Salud, UNAM, Mexico City 04510, Mexico; lmarquez@ib.unam.mx; 7 Centro Nacional de Investigación Disciplinaria en Salud Animal e Inocuidad, Instituto Nacional de Investigaciones Forestales, Agrícolas y Pecuarias, Mexico City 04010, Mexico

**Author Name and Order**

In the original publication [1], Rocío Lara-Romero’s name was not included in full, and this was corrected. The order of authors was modified, placing Rocío Lara-Romero first in the list of authors and José Francisco Rivera-Benítez last. Through the official provision of the Secretariat of Science, Humanities, Technology and Innovation of Mexico, through the Directorate of the National System of Researchers and its Honorable General Council, it was resolved that Rocío Lara-Romero demonstrated that she participated significantly in the creation of the published article, and according to her request, the order of the authors should be modified, placing her first in the list of authors. Author Contributions Statement appears here: Conceptualization, J.F.R.-B., J.D.l.L.-A., R.M.-B. and R.G.-M.; investigation, R.L.-R. and J.F.R.-B.; writing—original draft preparation, J.F.R.-B., R.L.-R., N.R.-M. and I.H.-C.; supervision, R.M.-B. and J.F.R.-B.; methodology, L.M.-V., R.L.-R., R.M.-B. and J.D.l.L.-A.; formal analysis, J.F.R.-B., R.L.-R., L.M.-V., N.R.-M. and I.H.-C.; project administration, J.F.R.-B.; funding acquisition, J.F.R.-B. All authors have read and agreed to the published version of the manuscript.

**Additional Affiliation(s)**

In the original publication [1], there was an error regarding the affiliation for Rocío Lara-Romero. The original affiliation should be replaced: Posgrado en Ciencias de la Producción y de la Salud Animal, Facultad de Estudios Superiores Cuautitlán, Universidad Nacional Autónoma de México, Mexico City 04510, Mexico.

All authors agree with these changes and state that the scientific conclusions are unaffected. This correction was approved by the Academic Editor. The original publication has also been updated.
