# Supplementary material for: Phylogenetic and Molecular Analysis of the Porcine Epidemic Diarrhea Virus in Mexico during the First Reported Outbreaks (2013–2017)
Source: Viruses. 2024 Feb 18;16(2):309. doi: 10.3390/v16020309 (PMC10891996; doi:10.3390/v16020309)
Supplement: Supplementary file 1 [file viruses-16-00309-s001.zip › viruses-2848967-supplementary.pdf]

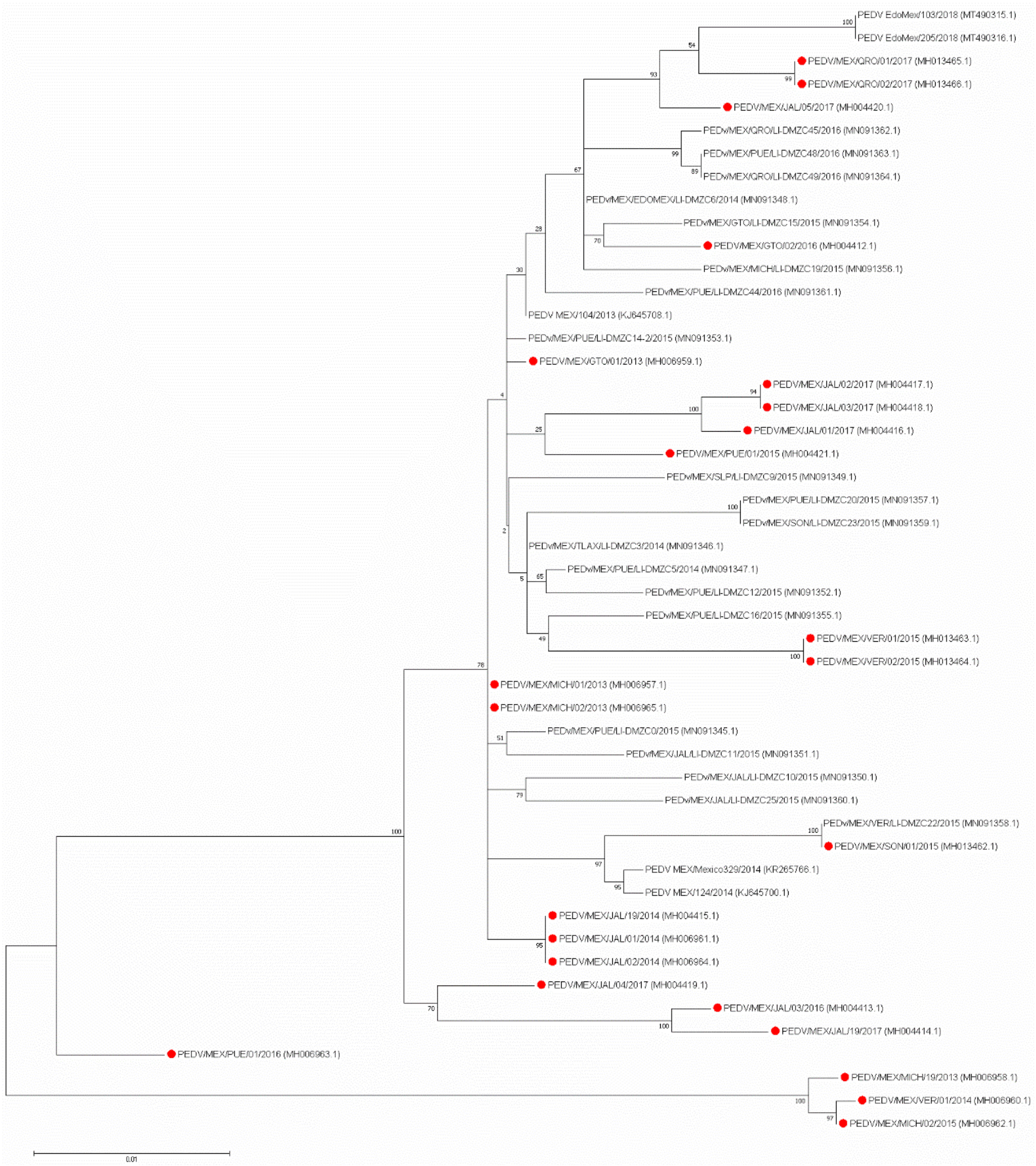

Supplementary Figure S1. Phylogenetic analyses of mexican PEDV spike protein sequences constructed by means of a maximum likelihood algorithm and 1000 bootstraps. The strains from this study are marked with a red circle. Phylogenetic tree of the S protein constructed with the model JTT+G+I (scale bar: 0.01).
